# Therapeutic Advances in Diabetic Nephropathy

**DOI:** 10.3390/jcm11020378

**Published:** 2022-01-13

**Authors:** Hanny Sawaf, George Thomas, Jonathan J. Taliercio, Georges Nakhoul, Tushar J. Vachharajani, Ali Mehdi

**Affiliations:** Department of Kidney Medicine, Glickman Urological & Kidney Institute, Cleveland Clinic Lerner College of Medicine, Cleveland Clinic, Cleveland, OH 44195, USA; SAWAFH@ccf.org (H.S.); thomasg3@ccf.org (G.T.); TALIERJ@ccf.org (J.J.T.); NAKHOUG@ccf.org (G.N.); MEHDIA@ccf.org (A.M.)

**Keywords:** diabetes, nephropathy, diabetic kidney disease, therapeutics, SGLT2, MRA, gliptins, flozins, ACE inhibitors, ARB

## Abstract

Diabetic kidney disease (DKD) is the most common cause of end-stage kidney disease (ESKD) in the United States. Risk factor modification, such as tight control of blood glucose, management of hypertension and hyperlipidemia, and the use of renin–angiotensin–aldosterone system (RAAS) blockade have been proven to help delay the progression of DKD. In recent years, new therapeutics including sodium-glucose transport protein 2 (SGLT2) inhibitors, endothelin antagonists, glucagon like peptide-1 (GLP-1) agonists, and mineralocorticoid receptor antagonists (MRA), have provided additional treatment options for patients with DKD. This review discusses the various treatment options available to treat patients with diabetic kidney disease.

## 1. Introduction

Diabetes is the leading cause of chronic kidney disease (CKD) and end-stage kidney disease (ESKD) in the United States and worldwide [1,2].

**Figure 1:**
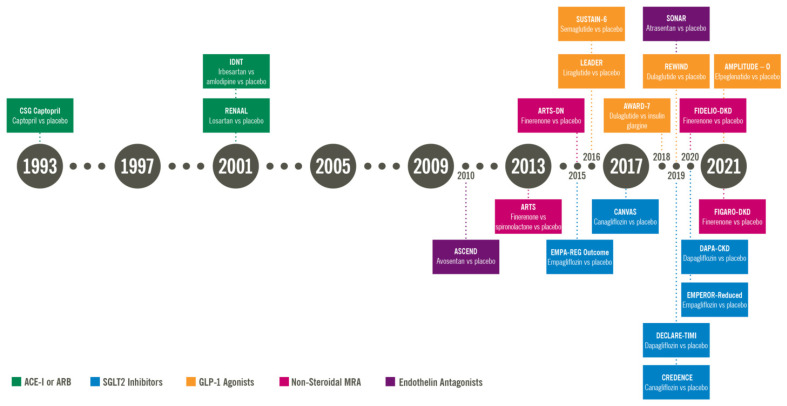
Timeline showing the landmark trials for diabetic kidney disease (DKD). The drug classes depected are angiotensin converting enzyme (ACE) inhibitors and angiotensin receptor blockers (ARB), sodium glucose transport protein 2 (SGLT2) inhibitors, glucagon like peptide 1 (GLP-1) agonists, non-steroidal mineralcorticoid receptor antagonists (MRA) and endothelin antagonists.

The renin–angiotensin–aldosterone system (RAAS) blockade, with angiotensin-converting enzyme inhibitors (ACE-I) and angiotensin receptor blockers (ARB), was the sole treatment option for diabetic kidney disease (DKD) for about 20 years. This review discusses the current landscape of treatment options for DKD, including an RAAS blockade with ACE-I or ARB, and newer therapies such as sodium-glucose transport protein 2 (SGLT2) inhibitors, endothelin antagonists, glucagon-like peptide-1 (GLP-1) agonists, and mineralocorticoid receptor antagonists (MRA), along with a summary of landmark trials that support the use of these agents (Figure 1).

## 2. Renin Angiotensin Aldosterone System (RAAS) Blockade (ACE-I and ARB) 

### 2.1. Introduction

**Figure 2:**
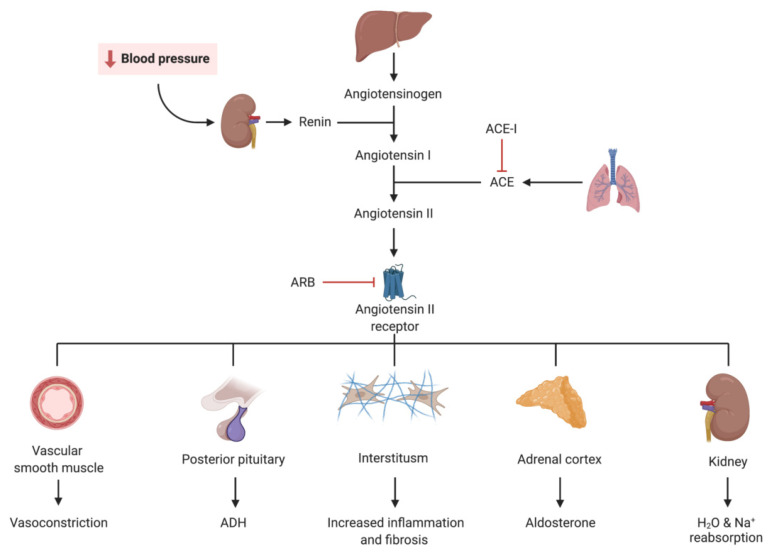
Visual representation of the renin–angiotensin–aldosterone system (RAAS) and the mechanism of action of angiotensin-converting enzyme inhibitors (ACE-I) and angiotensin receptor blockers (ARB).

The renin–angiotensin–aldosterone system is strongly linked with kidney and cardiac disease. The angiotensin-converting enzyme (ACE) converts angiotensin I to angiotensin II, which has pro-inflammatory and pro-fibrotic effects [3], increased sympathetic activity, increased tubular sodium and chloride absorption, increased aldosterone secretion, arteriolar vasoconstriction, and ADH secretion. ACE also plays a role in bradykinin metabolism [4] (Figure 2).

ACE inhibition and the angiotensin blockade in the form of ACE inhibitors and angiotensin receptor blockers have been proven to be useful in patients with kidney and cardiac diseases. The effects of angiotensin on kidney autoregulation was described as early as 1983 [5]. Over the following decade, several studies showed decreased albuminuria [6] and reno-protective effects with the RAAS blockade [7,8,9,10]. 

ACE-Is and ARBs have been well described in the literature, with landmark trials proving the therapeutic utility of this class of drugs for patients with DKD, and remain the mainstay of management of DKD (Table 1). 

### 2.2. Landmark Trials

The “CSG Captopril trial” compared captopril with placebo in patients with insulin-dependent diabetes mellitus, proteinuria ≥500 mg/day and creatinine ≤2.5 mg/dL, and showed that captopril was effective in slowing the deterioration of kidney function, independent of blood pressure control alone [11]. This paved the way for the subsequent RENAAL and IDNT trials.

The RENAAL trial (reduction of end-points in non-insulin-dependent diabetes mellitus with the angiotensin II antagonist losartan) randomized participants to losartan 50–100 mg/day vs placebo, in addition to conventional hypertension (HTN) therapy including calcium channel blockers, diuretics, alpha blockers, and beta blockers. The primary end-point of this study was the doubling of creatinine, ESKD, or death. The losartan arm had a 25% risk reduction for creatinine doubling, and a 28% risk reduction for ESKD but no effect on death [12]. 

The Irbesartan Diabetic Nephropathy Trial (IDNT) had three arms: irbesartan, amlodipine, and placebo, with a primary end-point of doubling creatinine, development of ESKD, and death. When compared with the placebo, irbesartan had a lower relative risk for the primary end-point, and also specifically for the doubling of creatinine [13]. 

With the benefits of RAAS inhibition firmly established, the next obvious question was whether combining ACE-I and ARB could provide an additional benefit. The ONTARGET trial, which compared telmisartan, ramipril, or both, in patients with high risk for vascular events, showed that a combined RAAS blockade with ACE-I and ARB was not associated with an improvement in outcome, but was associated with an increase in adverse events, including hypotensive episodes, syncope and renal impairment [14]. 

### 2.3. Practical Considerations

Although these medications have been used for decades for patients with DKD, there are some practical considerations to keep in mind when starting a patient on an ACE-I or ARB. 

When starting one of these medications, it is important to monitor the serum creatinine and potassium within 2–4 weeks after starting this medication or changing the dose. One should monitor for a medication-induced change in serum creatinine that is >30%, or a medication-induced hyperkalemia [15]. 

## 3. SGLT2 Inhibitors

### 3.1. Introduction

Initial studies with SGLT2 inhibitors explored their utility in improving blood sugar control in patients with type II diabetes mellitus (T2DM) by virtue of increased urinary glucose excretion [16,17]. 

**Figure 3:**
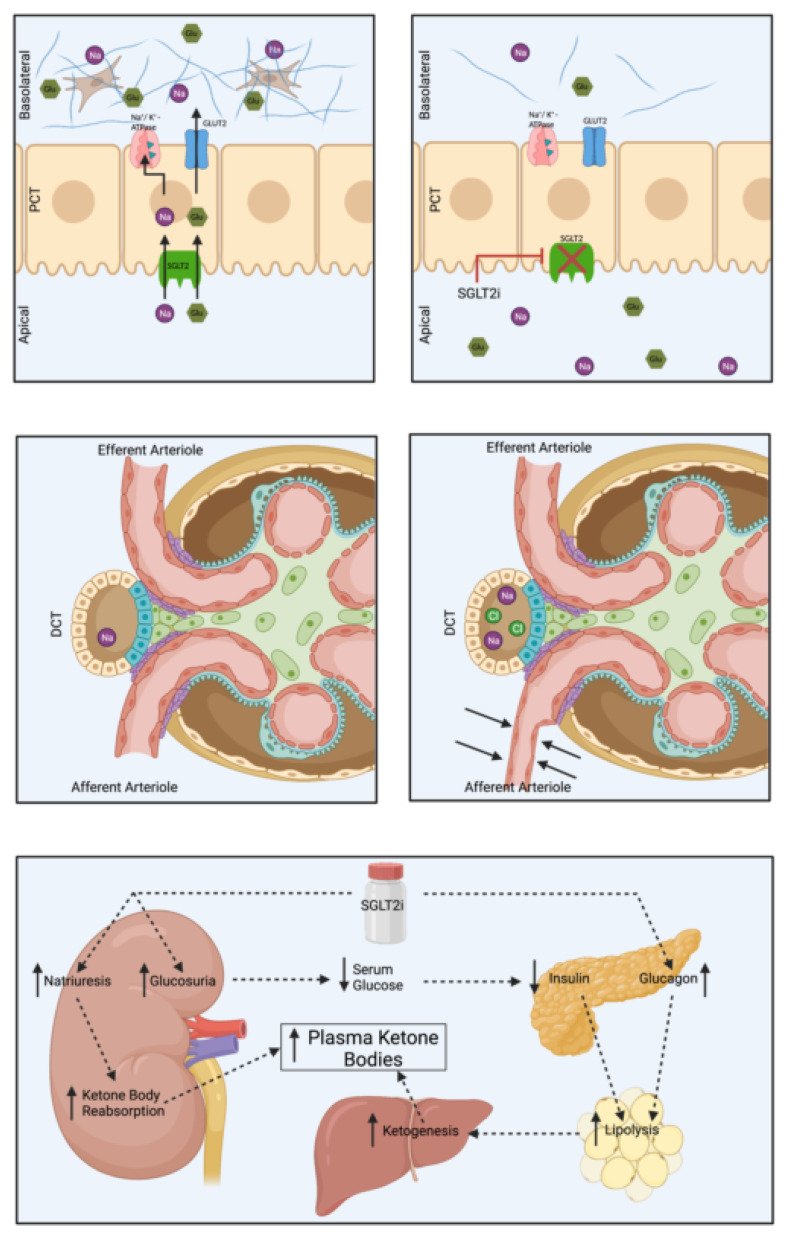
Visual representation of the mechanism of action of sodium glucose transport protein 2 (SGLT2) inhibitors. Top: Under normal circumstances (left), sodium and glucose are both reabsorbed by SGLT2 to enter the proximal tubular cell. The sodium then gets to the basolateral side of the proximal convoluted tubule (PCT) through the sodium–potassium exchanger, while glucose utilizes GLUT2 to exit the PCT cell. The presence of excessive glucose on the basolateral side of the PCT cell results in increased extracellular matrix production and fibrosis. Inhibiting SGLT2 will result in decreased sodium and glucose in the interstitial space (right), resulting in decreased extracellular matrix production and fibrosis. Middle: Blockade of SGLT2 results in increased sodium and chloride delivery to more distal portions of the nephron. This increased sodium and chloride is sensed by the macula densa, resulting in tubule-glomerular feedback which causes renal afferent arteriolar vasoconstriction. This vasoconstriction will decrease glomerular filtration and glomerular hypertension. Bottom: SGLT2 inhibitors will increase the production of plasma ketone bodies. The natriuresis caused by SGLT2 inhibitors will decrease the excretion of these plasma ketone bodies in the urine. These ketone bodies are preferentially oxidized over free fatty acids which play a role in a decreased oxidative stress on the kidneys and heart.

The beneficial effect of this class of medications are now known to go beyond their glucosuric effect, with independent cardiovascular and kidney benefits. Glomerular hypertension is one of the maladaptive mechanisms of the pathogenesis of DKD. The impact of SGLT2 inhibitors on this process remains the most widely accepted mechanism resulting in kidney protection. By blocking SGLT2 in the proximal tubule, SGLT2 inhibitors produce a natriuretic effect, which induces tubulo-glomerular feedback resulting in vasoconstriction in the afferent arteriole, reducing glomerular hyperfiltration [18]. The reduction in hyperfiltration, as with RAAS inhibitors, ultimately slows the progression of DKD (Figure 3).

Aside from their effect on glomerular pressure, it has also been hypothesized that excessive glucose uptake in the tubules can potentially contribute to proximal tubule production of extracellular matrix proteins, which may slow the progression of CKD [19]. In addition, studies have shown that SGLT2 inhibitors can simulate antioxidant and anti-inflammatory signaling pathways [20], which can potentially reverse molecular processes related to inflammation, extracellular matrix turnover, and fibrosis [21]. 

Another hypothesis is that SGLT2 inhibitors can induce a low ketotic state through the increased production of ketone bodies and their decreased urinary excretion. This “thrifty substrate” theory hypothesizes that the level of ketones can be preferentially oxidized over free fatty acids, which play a role in the decreased oxidative stress on the kidneys and heart [22,23].

Numerous trials have now established the cardiovascular and kidney benefits of SGLT2 inhibitors, and solidified their role in the management of patients with DKD and, most recently, proteinuric CKD in general (Table 2). 

### 3.2. Landmark Trials

The landmark trials involving SGLT2 inhibitors were initially designed to evaluate cardiovascular (CV) benefits, and their kidney benefits soon became apparent. In trials examining kidney benefits, patients were on clinically appropriate doses of RAAS inhibition, in the form of ACE-I or ARB, prior to being started on SGLT2 inhibitors. 

The CANVAS trial randomized patients to canagliflozin or placebo groups; the primary outcomes were death from CV causes, nonfatal myocardial infarction (MI) and nonfatal stroke. Amongst the patients in this study, 17.2% had a history of DKD, and in the final analysis there was reduction in the kidney composite outcome (reduction in eGFR, kidney replacement therapy, or kidney death); hazard ratio HR 0.60 (95% CI of 0.47–0.77). The hazard ratio for progression of albuminuria was also statistically significant at 0.73 (95% CI of 0.67–0.79) [25].

In order to further investigate the potential kidney benefits of canagliflozin, the CREDENCE trial examined kidney outcomes as the primary end-point in patients with albuminuric CKD, randomized to canagliflozin or placebo. The average eGFR was 56.2 mL/min/1.73m^2^ and the median urinary albumin–creatinine ratio (UACR) was 927 mg/g. The primary outcome, defined as ESKD, a doubling of the serum creatinine or death from kidney or cardiovascular cause, was lower in the canagliflozin group (HR 0.70; 95% CI 0.59–0.82) [26].

The Dapagliflozin Effect on Cardiovascular Events trial (DECLARE-TIMI 58 trial) was designed to further investigate kidney and cardiovascular outcomes related to dapagliflozin. This trial randomized its patients to receive either dapagliflozin or placebo. The kidney composite was the secondary end-point in this trial, and was defined as a ≥ 40% decrease in eGFR to <60 mL/min/1.73 m^2^, ESKD or death from kidney or cardiovascular cause. The hazard ratio for the renal composite was 0.76 (95% CI 0.67–0.87). The HR with individual components of renal composite improved even further to 0.53 (95% CI 0.43–0.66) [27].

The Dapagliflozin in Patients with Chronic Kidney Disease (DAPA-CKD) trial randomized patients with an eGFR of 25–75 mL/min/1.73 m^2^ and a UACR of 200–5000 mg/g to receive dapagliflozin or placebo, and was designed to investigate the long-term efficacy and safety profile of dapagliflozin in patients with CKD, with or without T2DM. The primary outcome in this trial was a composite of a sustained decline in the eGFR of at least 50%, ESKD, or death from kidney or cardiovascular cause. In this trial, only 67% of the patients had DM, and the trial showed a significantly lower risk for the primary outcome in patients with CKD, irrespective of whether they had DM. The primary composite outcome had an HR of 0.61 (95% CI 0.51–0.72) [28]. Study results were consistent across prespecified analysis of participants stratified by diabetes status, eGFR cutoff of 45 mL/min/1.73 m^2^, and UACR of 1 g/g. 

The EMPA-REG OUTCOME trial compared empagliflozin to placebo in patients with type 2 diabetes and a high risk for cardiovascular events, and had a primary composite outcome of death from CV causes, nonfatal MI, or nonfatal stroke [24]. In a separate report reviewing the kidney outcomes of this trial, the rate of doubling of serum creatinine level accompanied by an eGFR ≤45 mL/min/1.73 m^2^ initiation of kidney replacement therapy, or death from renal cause, was found to be lower with empagliflozin (HR 0.54; 95% CI 0.40–0.75) [30].

The EMPEROR-Reduced trial assigned patients with classes II–IV heart failure to receive empagliflozin or placebo. Composite renal outcome was one of the secondary outcomes, and this was defined as the need for chronic dialysis or kidney transplant, or a ≥40% decrease in eGFR, or a sustained eGFR <15 mL/min/1.73 m^2^ (if baseline eGFR was ≥ 30 mL/min/1.73 m^2^), or <10 mL/min/1.73 m^2^ (if the baseline eGFR was <30 mL/min/1.73 m^2^). Patients with an eGFR <20 mL/min/1.73 m^2^ at the time of randomization were excluded from the trial. The composite kidney outcome had an HR of 0.50 (95% CI 0.32–0.77) [29].

The EMPA-KIDNEY trial is an ongoing study with empagliflozin for patients with CKD for at least 3 months prior to screening, which was defined as an eGFR ≥20 to <45 mL/min/1.73 m^2^ of body surface area or an eGFR ≥45 to <90 mL/min/1.73 m^2^ with UACR ≥200 mg/g. The primary outcome is a composite of ESKD, sustained reduction in eGFR to <10 mL/min/1.73 m^2^, kidney death or a sustained decline of ≥ 40% in eGFR from randomization. The secondary outcomes in this study include CV death, hospitalizations, and all-cause mortality [31].

### 3.3. Practical Considerations

With the rapidly increasing utilities of SGLT2 inhibitors, there are numerous things to keep in mind. 

The increased rate of genital infection caused by SGLT2 inhibitors can be addressed by education on hygiene. Peri-area cleaning after urination and before sleeping can help decrease the risk of genital infections and UTIs. Patients should also be educated to seek help if they experience genital itching. 

Given the diuretic effect of SGLT2 inhibitors, a “sick day protocol” can be implemented where the medication is held while a patient is ill, especially if they are experiencing symptoms such as nausea, vomiting or diarrhea. If a patient is already on a diuretic, decreasing the dose of the diuretic can also be considered at the time of SGLT2 inhibitor initiation to decrease the chance of volume depletion. 

Although SGLT2 inhibitors do not cause hypoglycemia, they can lead to hypoglycemia when the patient is on another agent such as insulin or a sulfonylurea; therefore, decreasing the doses of insulin and/or sulfonylurea could be appropriate when adding SGLT2 inhibitors.

Finally, given the increased risk of diabetic keto acidosis (DKA), these medications should be approached with caution in patients with a history of DKA. When the medication is started, patients should be educated for the early recognition of this process.

## 4. GLP-1 Agonists

### 4.1. Introduction

Glucagon-like peptide-1 are incretin hormones secreted from L cells in the lower gut in response to food intake and an increase in plasma glucose. These incretin hormones facilitate insulin secretion from pancreatic ß islet cells, suppress glucagon secretion, delay gastric emptying, and induce a feeling of satiety [32,33]. 

In addition to the pancreas, GLP-1 receptors are found in a variety of organs, including but not limited to the lungs, stomach and kidneys [34,35]. 

**Figure 4:**
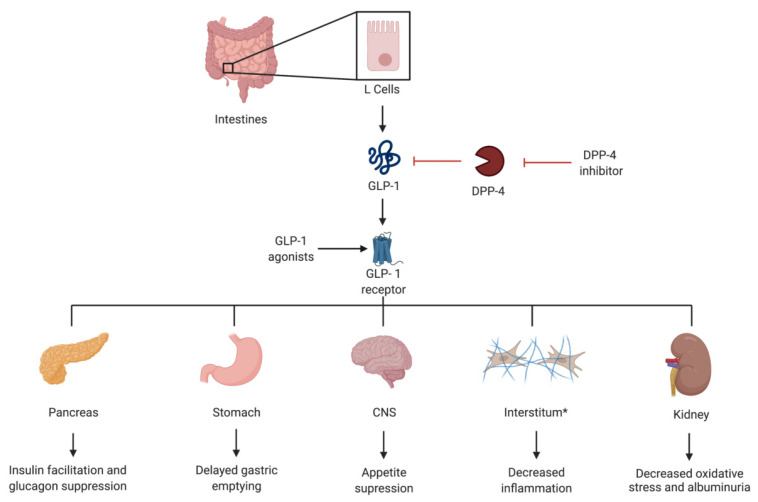
Visual representation of the mechanism of action of glucagon-like peptide 1 (GLP-1), as well as GLP-1 agonists and dipeptidyl peptidase-4 (DDP-4) inhibitors. * The anti-inflammatory effect has been noted in the heart and kidneys.

GLP-1 has also shown to have numerous kidney protective effects, including the inhibition of the inflammatory effects of angiotensin II [36] and the inhibition of oxidative stress and albuminuria [37], as well as an ability to ameliorate albuminuria, glomerular hyperfiltration, glomerular hypertrophy and mesangial matrix expansion in animal models [38] (Figure 4).

The initial use of GLP-1 agonism was as a supplemental agent to assist with the control of T2DM, and it was often used as an adjunct to other agents. Its weight loss properties provided an additional benefit, but recent trials have further demonstrated cardiovascular and kidney benefits, making this medication a new potential option for earlier utilization in patients with DKD (Table 3). 

### 4.2. Landmark Trials

The initial trials for GLP-1 agonists evaluated cardiovascular effects, with recent studies examining kidney protective effects. 

The LEADER trial was an early study investigating GLP-1 outcomes that provided insights into potential kidney benefits. This trial randomized patients with T2DM and high CV risk into receiving liraglutide or placebo. Roughly 75% of these patients had an eGFR > 60 mL/min/m^2^. The primary outcome in this trial was the cardiovascular outcome. Secondary microvascular events evaluating nephropathy was lower in the liraglutide arm (HR 0.78; 95% CI 0.67–0.92). This trial also showed that the group with eGFR of 30–59 mL/min/m^2^ had an HR for the primary end-point of 0.67 (95% CI 0.54–0.83), whereas those with eGFR < 30 mL/min/m^2^ and eGFR ≥ 60 mL/min/m^2^ did not have a statistically significant difference in the primary end-point, when compared with the placebo [39].

The SUSTAIN-6 trial compared semaglutide to placebo in patients with T2DM. The primary outcome in this study was CV death, non-fatal MI, or nonfatal stroke. One of the secondary outcomes—“new or worsening nephropathy”—included macroalbuminuria, persistent doubling of the serum creatinine, a creatinine clearance less than 45 mL/min/m^2^ or the need for dialysis, and was lower in the semaglutide group (HR 0.64; 95% CI 0.46–0.88) [40].

The first major randomized trial involving GLP-1 agonists that specifically evaluated patients with moderate-to-severe CKD was the AWARD-7 trial. A number of 3–4 CKD patients were randomized 1:1:1 to either receive dulaglutide at 0.75 mg weekly or 1.5 mg weekly, or to receive insulin glargine as basal therapy. Secondary outcomes included change in eGFR (calculated with the Chronic Kidney Disease Epidemiology Collaboration (CKD-EPI) equation by cystatin C and serum creatinine) and UACR. There was a significant decrease in the UACR with the high dose (1.5 mg) dulaglutide arm, when compared with the placebo. The trial also showed that the insulin arm experienced a statistically significant decrease in eGFR at 24 weeks, whereas the two dulaglutide arms kept eGFR similar to where it was at the start of the trial [41].

The REWIND trial was designed to assess high-risk cardiovascular patients with a broad range of glycemic control, with a weekly subcutaneous dose of dulaglutide versus placebo. The majority of these patients had an eGFR of >60 mL/min/m^2^. Kidney disease was one of the secondary outcomes investigated in this trial, defined as the development of a UACR > 300 mg/g in those with lower baseline concentrations, sustained 30% or a greater decline in eGFR or chronic need for KRT. Those randomized to the placebo arm had 4.07 kidney incidents per 100 person-years, compared with 3.47 kidney incidents per 100 person-years for those receiving dulaglutide (HR 0.85; 95% CI 0.77–0.93) [42].

A meta-analysis with a total of 56,004 participants from seven trials, including the LEADER [39], SUSTAIN-6 [40], and REWIND [42] trials, in addition to the ELIXA [45], EXSCEL [46], Harmony Outcomes [47] and the PIONEER 6 [48] trials, showed a 17% decrease in the composite renal outcome with GLP-1 agonists, with an HR of 0.83 (95% CI of 0.78–0.89) [43].

The AMPLITUDE-O trial compared efpeglenatide to placebo in patients with a history of CV disease or CKD, defined as an eGFR of 25.0–59.9 mL/min/1.73 m^2^. The trial included a composite kidney outcome, which was defined as incident microalbuminuria with a UACR of >300 mg/g, >30% UACR at baseline, a decrease in eGFR > 40% for 30 days or more, KRT > 90 days, or eGFR < 15 mL/min/1.73 m^2^ for >30 days. The composite kidney outcome in this trial occurred in 13% of those receiving efpeglenatide, versus 18.4% of those receiving placebo (HR 0.68; 95% CI 0.57–0.79) [44].

The FLOW trial is an ongoing DKD outcomes trial with semaglutide, where the primary end-point is a persistent 50% or more reduction in eGFR, an eGFR of less than 15 mL/min/1.73 m^2^, initiation of KRT or death from kidney disease and death from CV cause [49]. 

### 4.3. Practical Considerations

Given the frequency of gastrointestinal complications related to GLP-1 agonists such as nausea, vomiting and diarrhea, it is important to start these medications at lower doses and titrate slowly. The prescriber should also make sure patients being started on a GLP-1 agonist is aware of these effects as tolerabilities are being determined. Furthermore, it may be necessary to adjust other diabetic agents when a GLP-1 agonist is being initiated to avoid hypoglycemia. GLP-1 agonists should not be used in combination with dipeptidyl peptidase-4 (DDP-4) inhibitors. 

## 5. Mineralocorticoid Receptor Antagonists 

### 5.1. Introduction

Mineralocorticoid receptor (MR) mechanisms of action exceed beyond their antagonism on the epithelial sodium channels (ENaC) in the collecting tubules. MR are expressed in numerous tissues including colonic, cardiac and vascular tissues [50]. In addition to fluid and ion transport, MRs also play an important role in the adaptive response to injury [51]. Activation of these receptors has been shown to increase reactive oxygen species and inflammation [52], and overexpression of this receptor has been shown to lead to renal hypertrophy [53].

The earlier mineralocorticoid receptor antagonists that are still widely used are spironolactone and eplerenone. These steroidal MRAs were shown to protect from oxidative stress in animal models [54,55] and, in addition to being effective antihypertensive agents, landmark trials such as the RALES [56] and Emphasis-HF trials [57] proved their benefit and utility in patients with heart failure. Subsequent meta-analyses showed that these steroidal MRAs are effective agents in reducing proteinuria in patients already treated with an RAAS blockade [58,59]. Despite this potential renal benefit, these drugs remain underutilized in patients with CKD, given the concern for hyperkalemia [56,58,59,60] and worsening GFR [61].

The introduction of non-steroidal MRAs, with the most notable being finerenone, came with great promise for potentially providing cardiac and proteinuric benefits, while having a less profound effect on inducing hyperkalemia. Finerenone has numerous properties differentiating it from the steroidal MRAs, including its tissue distribution and the mode in which it causes MR inactivation, as well as other pharmacodynamic properties [61].

Finerenone is a welcome addition to our armamentarium, promising the same benefits of steroidal MRAs, but with less side effects. Numerous landmark trials have recently been published demonstrating the therapeutic potential of finerenone in DKD (Table 4). 

### 5.2. Landmark Trials

The Mineralocorticoid Receptor Antagonist Tolerability Study (ARTS) paved the way for future studies into finerenone. This study was divided into two parts, where part A was performed to evaluate the safety, tolerability and kidney effects of the drug, while part B included patients with moderate CKD. The study showed that finerenone was at least as effective as spironolactone in decreasing biomarkers for hemodynamic stress, while inducing less hyperkalemia and a decrease in renal function [62]. ARTS-DN trial, the follow up study, was designed to compare the efficacy and safety of varying doses of finerenone to placebo in patients with DKD and albuminuria. The study showed that there was a dose-dependent decrease in albuminuria in patients receiving finerenone when the dose was between 7.5 mg and 20 mg a day [63]. 

Building on the ARTS trials, the FIDELIO-DKD trial was a clinical outcomes study of finerenone compared to the placebo in patients with T2DM and CKD already treated with a maximal dose of ACE-I or ARB. The primary outcomes included kidney failure (defined as ESKD or an eGFR <15 mL/min/1.73 m^2^), >40% decrease from the baseline eGFR or death from kidney cause. The primary outcome was significantly lower in the finerenone group with an HR of 0.82 (95% CI 0.73–0.93). Hyperkalemia occurred in 11.8% of patients in the finerenone arm, compared with 4.8% of patients in the placebo arm [64].

The FIGARO-DKD went beyond what was investigated in the earlier FIDELIO-DKD trial, evaluating patients with T2DM with a boarder range of CKD stages. Patients in this study were grouped into eGFR 25-90 mL/min/1.73m2 with UACR of 30–300 mg/g and an eGFR≥ 60 mL/min/1.73m^2^ and UACR of 300-5000 mg/g. The composite outcome of kidney failure, a decrease of at least 40% in eGFR and death from kidney cause was the secondary outcome in this trial. The composite kidney outcome was 9.5% in the finerenone group, and 10.8% of the placebo group with an HR of 0.87 (95% CI 0.76–1.01) [65].

### 5.3. Practical Considerations

Hyperkalemia remains a side effect that a prescriber should be aware of when starting finerenone. Other common side effects include hypotension and hyponatremia. 

## 6. Endothelin Antagonists

### 6.1. Introduction

Endothelins comprise three structurally similar peptides involved in various vasoconstrictor pathways [66]. The kidney expresses endothelin receptors [67], and there is evidence of their overexpression in diabetics. The renal endothelin system has been shown to play an important role in normal renal function, and derangements of this system are involved in the initiation and progression of DKD, HTN and even glomerular nephritis [68].

Endothelin receptor antagonism has been shown to improve kidney microcirculation [69], and there has been evidence that endothelin antagonism can lower urinary protein excretion [70]. 

Given this evidence of potential kidney benefit, trials have been conducted to assess the benefits of endothelin antagonists in the treatment of patients with DKD (Table 5). 

### 6.2. Landmark Trials

The ASCEND trial randomized patients to receive either avosentan or placebo, and the primary outcome of the trial was a kidney composite which included the doubling of serum creatinine, ESKD, or death. The trial was terminated early due to safety concerns related to volume overload and congestive heart failure. Although there was a statistically significant reduction in the UACR in patients in the avosentan arm, there was no statistically significant difference in the kidney composite [71].

The SONAR trial was designed to evaluate whether endothelin antagonists could have a role in certain populations with DKD. In this trial, patients with T2DM, an eGFR of 25–75 mL/min/1.73 m^2^ and a UACR of 300–5000 mg/g on maximal tolerated RAAS blockade for 4 weeks were initially treated with atrasentan during the enrichment period. Patients who tolerated atrasentan without substantial volume retention were then randomized to receive atrasentan or placebo. The primary end-point was a kidney composite which included the doubling of serum creatinine for ≥ 30 days, or ESKD in the intent to treat population. The trial showed that the patients who tolerated the endothelin antagonist had improved kidney outcomes with HR of 0.65 (CI 95% 0.49–0.88). The trial did show higher amounts of fluid retention and anemia in the artesentan arm. Moreover, higher hospitalization for heart failure was seen in the atresentan arm (3.5% vs. 2.6% in the placebo arm), although the difference was not statistically significant [72].

### 6.3. Practical Considerations

Given the early termination of the ASCEND trial and the “enrichment period” performed in the SONAR trial, it is important to monitor for signs of volume overload should a patient be started on an endothelin agonist.

## 7. Potential Future Therapeutic Options

In addition to GLP-1 agonists, there is early evidence suggesting DDP-4 inhibitors could provide benefit for patients with DKD. Trials such as SAVOR-TIMI 53 [73] and CARMELINA [74] showed a possible reduction in albuminuria in patients receiving DPP-4 inhibitors. 

Given the role various inflammatory pathways have been shown to play in the progression of DKD, pharmacologic intervention targeting these pathways have been areas of interest for potential treatment approaches. One promising pathway involves JAK1/JAK2 inhibition by baricitinib. This has been shown to decrease albuminuria; however, it remains unclear how it would affect the progression of DKD [75]. Other potential targets include antifibrotic therapy with pirfenidone or pentoxifyolline [76], Nox1/4 inhibition [77,78] and chemokine cytokine inhibition [79,80]. Further investigation and clinical trials will show whether these innovative therapeutic interventions will eventually make it into our expanding repertoire of management options for DKD. 

The future of DKD management has the potential to include a more personalized approach, where each patient can have a tailored treatment regimen based on their genetic and biomarker profile [81]. 

## 8. Summary

For years, RAAS inhibition with ACE-I and ARBs was the sole therapeutic option we could offer patients with DKD. These agents certainly remain the cornerstone for managing these patients, but we are now fortunate to be able to offer other agents which can complement the benefit of the RAAS blockade. 

SGLT2 inhibitors are taking the nephrology world by storm with unequivocal protective benefits that extend beyond DKD. GLP-1 agonists are now preferred oral agents for the managements of diabetes in CKD patients, according to the inaugural KDIGO guidelines [15]. Finerenone is also a very welcome addition to our growing armamentarium, as we get closer to a guideline directed medical therapy for DKD, which would include an ACE-I or ARB, MRA, SGLT2 inhibitors, and possibly a GLP-1 agonist. We are fortunate to be living in an era where we are able to offer intervention, not only to slow down the progression of DKD, but also prevent cardiovascular complications and improve survival. 

These expanded therapeutic options have ushered in a new era of DKD management, enabling, cardiovascular, kidney, and survival benefits.

## Figures and Tables

**Table 1:** Summaries of landmark trials with the RAAS blockade.

Trial	Publication Year	Treatment(s)	Primary Composite Kidney Outcome	Risk Reduction
CSG Captopril [11]	1993	Captopril vs. placebo	Doubling of the base-line serum creatinine concentration	48%
RENAAL [12]	2001	Losartan vs. placebo	Doubling of serum creatinine, ESKD or death	16%
IDNT [13]	2001	Irbesartan vs. amlodipine vs. placebo	Doubling of serum creatinine, ESKD or death	20% vs. placebo 23% vs. amlodipine

CSG Captopril = the collaborative study group; ESKD = end-stage kidney disease; IDNT = Irbesartan Diabetic Nephropathy Trial; RENAAL = reduction of end-points in non-insulin-dependent diabetes mellitus with the angiotensin II antagonist losartan.

**Table 2:** Summaries of landmark trials with SGLT2 inhibitors.

Trial	Year Published	Treatment (s)	Primary or Secondary End-Point	Composite Kidney Outcome	Hazard Ratio (95% CI)
EMPA-REG OUTCOME [24]	2015	Empagliflozin vs. placebo	Secondary	Doubling of serum creatinine, initiation of kidney replacement therapy or death from renal disease	0.54 (0.40–0.75)
CANVAS [25]	2017	Canagliflozin vs. placebo	Secondary	Sustained 40% reduction in eGFR, need for kidney replacement therapy, or death from renal cause	0.6 (0.47–0.77)
CREDENCE [26]	2019	Canagliflozin vs. placebo	Primary	End-stage kidney disease, doubling of the serum creatinine level, or death from renal or cardiovascular causes	0.70 (0.59– 0.82)
DECLARE-TIMI [27]	2019	Dapagliflozin vs. placebo	Secondary	Sustained ≥40% reduction in eGFR to <60 mL/min/1.73 m^2^, new end-stage kidney disease or death from renal cause	0.53 (0.43–0.66)
DAPA-CKD [28]	2020	Dapagliflozin vs. placebo	Primary	Sustained ≥50% reduction in eGFR, end-stage kidney disease, or death from renal or cardiovascular cause	0.61 (0.51–0.72)
EMPEROR-Reduced [29]	2020	Empagliflozin vs. placebo	Secondary	Sustained ≥40% reduction in eGFR, chronic dialysis, renal transplant or sustained eGFR < 10–15 mL/min/1.73 m^2^	0.50 (0.32–0.77)
EMPA-KIDNEY	2022	Empagliflozin vs. placebo	Primary	End-stage kidney disease, a sustained reduction in eGFR to <10 mL/min/1.73 m^2^, renal death, or a sustained decline of ≥40% in eGFR	Ongoing

CANVAS = Canagliflozin Cardiovascular Assessment Study; CI = confidence interval; CREDENCE = Canagliflozin and Renal Events in Diabetes with Established Nephropathy Clinical Evaluation; DAPA-CKD = Dapagliflozin in patients with chronic kidney disease; DECLARE-TIMI = Dapagliflozin effect on cardiovascular events; eGFR = estimated glomerular filtration rate; EMPA-KIDNEY = study of heart and kidney protection with empagliflozin; EMPA-REG OUTCOME = empagliflozin cardiovascular outcome event trial in type 2 diabetes mellitus patients; EMPEROR-Reduced = Empagliflozin Outcome Trial in patients with chronic heart failure with reduced ejection fraction.

**Table 3:** Summaries of landmark trials with GLP-1 agonists.

Trial	Year Published	Treatment (s)	Primary or Secondary	Kidney Outcome	Results
LEADER [39]	2016	Liraglutide vs. placebo	Secondary	Diabetic Nephropathy	HR 0.78 (95% CI 0.67–0.92)
SUSTAIN-6 [40]	2016	Semaglutide vs. placebo	Secondary	Macroalbuminuria, doubling of serum creatinine, Creatinine clearance ≤ 45 mL/min or KRT	HR 0.64 (95% CI 0.46–0.88)
AWARD-7 [41]	2018	Dulaglutide vs. insulin glargine	Secondary	eGFR and UACR	A decline in eGFR of the insulin arm but not in the higher-dose dulaglutide arm
REWIND [42]	2019	Dulaglutide vs. placebo	Secondary	300 mg/g > UACR in lower baseline concentration, sustained 30% > eGFR decline, KRT	HR 0.85 (95% CI 0.77–0.93)
Kristensen et. al. meta-analysis [43]	2019	GLP-1′s	---	New-onset macroalbuminuria, decline in eGFR, progression of kidney disease or death of kidney cause	HR 0.83 (95% CI 0.78–0.89)
AMPLITUDE-O [44]	2021	Efpeglenatide vs. placebo	Secondary	Incident microalbuminuria > 300 mg/g, increase in UACR of at least 30% from baseline, sustained eGFR decrease > 40% for > 30 days, KRT for 90 days or more, eGFR < 15 for 30 days or more	HR 0.68 (95% CI 0.57–0.79)
FLOW	To be completed in 2024	Semaglutide vs. placebo	Primary	Persistent ≥ 50% reduction in eGFR, reaching ESKD, death from kidney disease or death from CV cause	Ongoing

AMPLITUDE-O = cardiovascular and renal outcomes with efpeglenatide in type 2 diabetes; AWARD-7 = dulaglutide versus insulin glargine in patients with type 2 diabetes and moderate-to-severe chronic kidney disease; CI = confidence interval; CKRT = continuous kidney replacement therapy; CV = cardiovascular; FLOW = effect of semaglutide versus placebo on the progression of renal impairment in subjects with type 2 diabetes and chronic kidney disease; eGFR = estimated glomerular filtration rate; ESKD = end-stage kidney disease; HR = hazard ratio; KRT = kidney replacement therapy; LEADER = liraglutide effect and action in diabetes: evaluation of cardiovascular outcome results; REWIND = dulaglutide and cardiovascular outcomes in type 2 diabetes; SUSTAIN-6 = trial to evaluate cardiovascular and other long-term outcomes with semaglutide in subjects with type 2 diabetes; UACR = urine albumin to creatinine ratio.

**Table 4:** Summarizes landmark trials with MRAs.

Trial	Year Published	Composite Kidney Outcome	Primary or Secondary End-Point	Findings or Results
ARTS [62]	2013	Change in serum potassium	Primary	Significant increases in potassium concentrations at 10 mg/day or more
		Effect eGFR	Secondary	No change in renal impairment
ARTS-DN [63]	2015	Change in UACR	Primary	Dose dependent placebo-corrected mean UACR
		Potassium and eGFR safety points	Secondary	1.7–3.2% discontinuation for hyperkalemia in finerenone arm No finerenone discontinuation due to drop in eGFR
FIDELIO-DKD [64]	2020	Kidney failure, >40% decrease in eGFR, death from kidney cause	Primary	HR 0.82 (95% CI 0.73–0.93)
FIGARO-DKD [65]	2021	Kidney failure, >40% decrease in eGFR, death from kidney cause	Secondary	HR 0.87 (95% CI 0.76–1.01)

ARTS = Mineralocorticoid Receptor Antagonist Tolerability study; ARTS-DN = Mineralocorticoid Receptor Antagonist Tolerability Study Diabetic Nephropathy; CI = confidence interval; eGFR = estimated glomerular filtration rate; FIDELIO-DKD = The finerenone in reducing kidney failure and disease progression in diabetic kidney disease; FIGARO-DKD = finerenone in reducing cardiovascular mortality and morbidity in diabetic kidney disease; HR = hazard ratio; UACR = urine albumin to creatinine ratio.

**Table 5:** Summaries of landmark trials with endothelin receptor blockers.

Trial	Year	Kidney Outcomes	Findings	Notes
ASCEND [71]	2010	Doubling of serum creatinine, ESKD, death	No significant change in primary outcome composite	Trial ended early due to safety concerns related to volume overload and CHF
SONAR [72]	2019	Doubling of serum creatinine, ESKD	HR 0.65 (CI 95% 0.49–0.88)	Trial included and “enrichment period” to determine who can tolerate endothelin antagonist prior to randomization

ASCEND = A randomized, double-blind, placebo-controlled, parallel group study to assess the effect of the endothelin receptor antagonist avosentan on time to doubling of serum creatinine, end-stage renal disease, or death, in patients with type 2 diabetes mellitus and diabetic nephropathy; CHF = congestive heart failure; CI = confidence interval; ESKD = end-stage kidney disease; HR = hazard ratio; SONAR = Study of Diabetic Nephropathy with Atrasentan.

## Data Availability

Data sharing not applicable. No new data were created or analyzed in this study. Data sharing is not applicable to this article.

## Abbreviations

ACE-I	angiotensin converting enzyme inhibitor
ARB	Angiotensin receptor blocker
CHF	Congestive heart failure
CKD	Chronic kidney disease
CKRT	continuous kidney replacement therapy
CI	confidence interval
CV	cardiovascular
DKA	diabetic keto acidosis
DKD	diabetic kidney disease
ESKD	end stage kidney disease
eGFR	estimated glomerular filtration rate
ENaC	Epithelial sodium channel
GLP-1	glucagon like peptide-1
HR	Hazard ratio
HTN	Hypertension
KRT	Kidney replacement therapy
MI	myocardial infarction
MR	mineralocorticoid receptor
MRA	mineralocorticoid receptor antagonist
RAAS	renin–angiotensin–aldosterone system
SGLT2	sodium-glucose transport protein 2
T2DM	type II diabetes mellitus
UACR	urine albumin to creatinine ratio

## References

[1] United States Renal Data System (2017). USRDS 2018 Annual Data Report: Atlas of Chronic Kidney Disease and End-Stage Renal Disease in the United States.

[2] International Diabetes Federation (2017). IDF Diabetes Atlas.

[3] Ames M.K., Atkins C.E., Pitt B. (2019). The renin-angiotensin-aldosterone system and its suppression. J. Vet. Intern. Med..

[4] Montinaro V., Cicardi M. (2020). ACE inhibitor-mediated angioedema. Int. Immunopharmacol..

[5] Blythe W.B. (1983). Captopril and renal autoregulation. N. Engl. J. Med..

[6] Hommel E., Parving H.H., Mathiesen E., Edsberg B., Damkjaer Nielsen M., Giese J. (1986). Effect of captopril on kidney function in insulin-dependent diabetic patients with nephropathy. Br. Med. J..

[7] Björck S., Nyberg G., Mulec H., Granerus G., Herlitz H., Aurell M. (1986). Beneficial effects of angiotensin converting enzyme inhibition on renal function in patients with diabetic nephropathy. Br. Med. J..

[8] Lagrue G., Robeva R., Laurent J. (1987). Antiproteinuric effect of captopril in primary glomerular disease. Nephron.

[9] Ikeda T., Nakayama D., Gomi T., Sakurai J., Yamazaki T., Yuhara M. (1989). Captopril, an angiotensin I-converting enzyme inhibitor, decreases proteinuria in hypertensive patients with renal diseases. Nephron.

[10] Heeg J.E., de Jong P.E., van der Hem G.K., de Zeeuw D. (1987). Reduction of proteinuria by angiotensin converting enzyme inhibition. Kidney Int..

[11] Lewis E.J., Hunsicker L.G., Bain R.P., Rohde R.D. (1993). The Effect of Angiotensin-Converting-Enzyme Inhibition on Diabetic Nephropathy. N. Engl. J. Med..

[12] Brenner B.M., Cooper M.E., de Zeeuw D., Keane W.F., Mitch W.E., Parving H.H., Remuzzi G., Snapinn S.M., Zhang Z., Shahinfar S. (2001). Effects of losartan on renal and cardiovascular outcomes in patients with type 2 diabetes and nephropathy. N. Engl. J. Med..

[13] Lewis E.J., Hunsicker L.G., Clarke W.R., Berl T., Pohl M.A., Lewis J.B., Ritz E., Atkins R.C., Rohde R., Raz I. (2001). Renoprotective effect of the angiotensin-receptor antagonist irbesartan in patients with nephropathy due to type 2 diabetes. N. Engl. J. Med..

[14] Yusuf S., Teo K.K., Pogue J., Dyal L., Copland I., Schumacher H., Dagenais G., Sleight P., Anderson C. (2008). Telmisartan, ramipril, or both in patients at high risk for vascular events. N. Engl. J. Med..

[15] de Boer I.H., Caramori M.L., Chan J.C.N., Heerspink H.J.L., Hurst C., Khunti K., Liew A., Michos E.D., Navaneethan S.D., Olowu W.A. (2020). Executive summary of the 2020 KDIGO Diabetes Management in CKD Guideline: Evidence-based advances in monitoring and treatment. Kidney Int..

[16] Sha S., Devineni D., Ghosh A., Polidori D., Chien S., Wexler D., Shalayda K., Demarest K., Rothenberg P. (2011). Canagliflozin, a novel inhibitor of sodium glucose co-transporter 2, dose dependently reduces calculated renal threshold for glucose excretion and increases urinary glucose excretion in healthy subjects. Diabetes Obes. Metab..

[17] Devineni D., Morrow L., Hompesch M., Skee D., Vandebosch A., Murphy J., Ways K., Schwartz S. (2012). Canagliflozin improves glycaemic control over 28 days in subjects with type 2 diabetes not optimally controlled on insulin. Diabetes Obes. Metab..

[18] Fioretto P., Zambon A., Rossato M., Busetto L., Vettor R. (2016). SGLT2 Inhibitors and the Diabetic Kidney. Diabetes Care.

[19] Gilbert R.E. (2014). Sodium-glucose linked transporter-2 inhibitors: Potential for renoprotection beyond blood glucose lowering?. Kidney Int..

[20] Hasan R., Lasker S., Hasan A., Zerin F., Zamila M., Parvez F., Rahman M.M., Khan F., Subhan N., Alam M.A. (2020). Canagliflozin ameliorates renal oxidative stress and inflammation by stimulating AMPK-Akt-eNOS pathway in the isoprenaline-induced oxidative stress model. Sci. Rep..

[21] Heerspink H.J.L., Desai M., Jardine M., Balis D., Meininger G., Perkovic V. (2017). Canagliflozin Slows Progression of Renal Function Decline Independently of Glycemic Effects. J. Am. Soc. Nephrol..

[22] Ferrannini E., Mark M., Mayoux E. (2016). CV Protection in the EMPA-REG OUTCOME Trial: A “Thrifty Substrate” Hypothesis. Diabetes Care.

[23] Mudaliar S., Alloju S., Henry R.R. (2016). Can a Shift in Fuel Energetics Explain the Beneficial Cardiorenal Outcomes in the EMPA-REG OUTCOME Study? A Unifying Hypothesis. Diabetes Care.

[24] Zinman B., Wanner C., Lachin J.M., Fitchett D., Bluhmki E., Hantel S., Mattheus M., Devins T., Johansen O.E., Woerle H.J. (2015). Empagliflozin, Cardiovascular Outcomes, and Mortality in Type 2 Diabetes. N. Engl. J. Med..

[25] Neal B., Perkovic V., Mahaffey K.W., de Zeeuw D., Fulcher G., Erondu N., Shaw W., Law G., Desai M., Matthews D.R. (2017). Canagliflozin and Cardiovascular and Renal Events in Type 2 Diabetes. N. Engl. J. Med..

[26] Perkovic V., Jardine M.J., Neal B., Bompoint S., Heerspink H.J.L., Charytan D.M., Edwards R., Agarwal R., Bakris G., Bull S. (2019). Canagliflozin and Renal Outcomes in Type 2 Diabetes and Nephropathy. N. Engl. J. Med..

[27] Wiviott S.D., Raz I., Bonaca M.P., Mosenzon O., Kato E.T., Cahn A., Silverman M.G., Zelniker T.A., Kuder J.F., Murphy S.A. (2019). Dapagliflozin and Cardiovascular Outcomes in Type 2 Diabetes. N. Engl. J. Med..

[28] Heerspink H.J.L., Stefánsson B.V., Correa-Rotter R., Chertow G.M., Greene T., Hou F.-F., Mann J.F.E., McMurray J.J.V., Lindberg M., Rossing P. (2020). Dapagliflozin in Patients with Chronic Kidney Disease. N. Engl. J. Med..

[29] Packer M., Anker S.D., Butler J., Filippatos G., Pocock S.J., Carson P., Januzzi J., Verma S., Tsutsui H., Brueckmann M. (2020). Cardiovascular and Renal Outcomes with Empagliflozin in Heart Failure. N. Engl. J. Med..

[30] Wanner C. (2017). EMPA-REG OUTCOME: The Nephrologist’s Point of View. Am. J. Med..

[31] Rhee J.J., Jardine M.J., Chertow G.M., Mahaffey K.W. (2020). Dedicated kidney disease-focused outcome trials with sodium-glucose cotransporter-2 inhibitors: Lessons from CREDENCE and expectations from DAPA-HF, DAPA-CKD, and EMPA-KIDNEY. Diabetes Obes. Metab..

[32] Tanaka T., Higashijima Y., Wada T., Nangaku M. (2014). The potential for renoprotection with incretin-based drugs. Kidney Int..

[33] Turton M.D., O’Shea D., Gunn I., Beak S.A., Edwards C.M., Meeran K., Choi S.J., Taylor G.M., Heath M.M., Lambert P.D. (1996). A role for glucagon-like peptide-1 in the central regulation of feeding. Nature.

[34] Bullock B.P., Heller R.S., Habener J.F. (1996). Tissue distribution of messenger ribonucleic acid encoding the rat glucagon-like peptide-1 receptor. Endocrinology.

[35] Campos R.V., Lee Y.C., Drucker D.J. (1994). Divergent tissue-specific and developmental expression of receptors for glucagon and glucagon-like peptide-1 in the mouse. Endocrinology.

[36] Mima A., Hiraoka-Yamomoto J., Li Q., Kitada M., Li C., Geraldes P., Matsumoto M., Mizutani K., Park K., Cahill C. (2012). Protective effects of GLP-1 on glomerular endothelium and its inhibition by PKCβ activation in diabetes. Diabetes.

[37] Hendarto H., Inoguchi T., Maeda Y., Ikeda N., Zheng J., Takei R., Yokomizo H., Hirata E., Sonoda N., Takayanagi R. (2012). GLP-1 analog liraglutide protects against oxidative stress and albuminuria in streptozotocin-induced diabetic rats via protein kinase A-mediated inhibition of renal NAD(P)H oxidases. Metabolism.

[38] Kodera R., Shikata K., Kataoka H.U., Takatsuka T., Miyamoto S., Sasaki M., Kajitani N., Nishishita S., Sarai K., Hirota D. (2011). Glucagon-like peptide-1 receptor agonist ameliorates renal injury through its anti-inflammatory action without lowering blood glucose level in a rat model of type 1 diabetes. Diabetologia.

[39] Marso S.P., Daniels G.H., Brown-Frandsen K., Kristensen P., Mann J.F.E., Nauck M.A., Nissen S.E., Pocock S., Poulter N.R., Ravn L.S. (2016). Liraglutide and Cardiovascular Outcomes in Type 2 Diabetes. N. Engl. J. Med..

[40] Marso S.P., Bain S.C., Consoli A., Eliaschewitz F.G., Jódar E., Leiter L.A., Lingvay I., Rosenstock J., Seufert J., Warren M.L. (2016). Semaglutide and Cardiovascular Outcomes in Patients with Type 2 Diabetes. N. Engl. J. Med..

[41] Tuttle K.R., Lakshmanan M.C., Rayner B., Busch R.S., Zimmermann A.G., Woodward D.B., Botros F.T. (2018). Dulaglutide versus insulin glargine in patients with type 2 diabetes and moderate-to-severe chronic kidney disease (AWARD-7): A multicentre, open-label, randomised trial. Lancet Diabetes Endocrinol..

[42] Gerstein H.C., Colhoun H.M., Dagenais G.R., Diaz R., Lakshmanan M., Pais P., Probstfield J., Riesmeyer J.S., Riddle M.C., Rydén L. (2019). Dulaglutide and cardiovascular outcomes in type 2 diabetes (REWIND): A double-blind, randomised placebo-controlled trial. Lancet.

[43] Kristensen S.L., Rørth R., Jhund P.S., Docherty K.F., Sattar N., Preiss D., Køber L., Petrie M.C., McMurray J.J.V. (2019). Cardiovascular, mortality, and kidney outcomes with GLP-1 receptor agonists in patients with type 2 diabetes: A systematic review and meta-analysis of cardiovascular outcome trials. Lancet Diabetes Endocrinol..

[44] Gerstein H.C., Sattar N., Rosenstock J., Ramasundarahettige C., Pratley R., Lopes R.D., Lam C.S.P., Khurmi N.S., Heenan L., Del Prato S. (2021). Cardiovascular and Renal Outcomes with Efpeglenatide in Type 2 Diabetes. N. Engl. J. Med..

[45] Pfeffer M.A., Claggett B., Diaz R., Dickstein K., Gerstein H.C., Køber L.V., Lawson F.C., Ping L., Wei X., Lewis E.F. (2015). Lixisenatide in Patients with Type 2 Diabetes and Acute Coronary Syndrome. N. Engl. J. Med..

[46] Holman R.R., Bethel M.A., Mentz R.J., Thompson V.P., Lokhnygina Y., Buse J.B., Chan J.C., Choi J., Gustavson S.M., Iqbal N. (2017). Effects of Once-Weekly Exenatide on Cardiovascular Outcomes in Type 2 Diabetes. N. Engl. J. Med..

[47] Hernandez A.F., Green J.B., Janmohamed S., D’Agostino R.B., Granger C.B., Jones N.P., Leiter L.A., Rosenberg A.E., Sigmon K.N., Somerville M.C. (2018). Albiglutide and cardiovascular outcomes in patients with type 2 diabetes and cardiovascular disease (Harmony Outcomes): A double-blind, randomised placebo-controlled trial. Lancet.

[48] Husain M., Birkenfeld A.L., Donsmark M., Dungan K., Eliaschewitz F.G., Franco D.R., Jeppesen O.K., Lingvay I., Mosenzon O., Pedersen S.D. (2019). Oral Semaglutide and Cardiovascular Outcomes in Patients with Type 2 Diabetes. N. Engl. J. Med..

[49] Williams D.M., Evans M. (2020). Semaglutide: Charting New Horizons in GLP-1 Analogue Outcome Studies. Diabetes Ther. Res. Treat. Educ Diabetes Relat Disord.

[50] Buonafine M., Bonnard B., Jaisser F. (2018). Mineralocorticoid Receptor and Cardiovascular Disease. Am. J. Hypertens.

[51] Gomez-Sanchez E., Gomez-Sanchez C.E. (2014). The multifaceted mineralocorticoid receptor. Compr. Physiol..

[52] Gomez-Sanchez E.P., Gomez-Sanchez C.E. (2012). Central regulation of blood pressure by the mineralocorticoid receptor. Mol. Cell Endocrinol..

[53] Le Menuet D., Isnard R., Bichara M., Viengchareun S., Muffat-Joly M., Walker F., Zennaro M.C., Lombès M. (2001). Alteration of cardiac and renal functions in transgenic mice overexpressing human mineralocorticoid receptor. J. Biol. Chem..

[54] Barrera-Chimal J., Pérez-Villalva R., Rodríguez-Romo R., Reyna J., Uribe N., Gamba G., Bobadilla N.A. (2013). Spironolactone prevents chronic kidney disease caused by ischemic acute kidney injury. Kidney Int..

[55] Mejía-Vilet J.M., Ramírez V., Cruz C., Uribe N., Gamba G., Bobadilla N.A. (2007). Renal ischemia-reperfusion injury is prevented by the mineralocorticoid receptor blocker spironolactone. Am. J. Physiol. Ren. Physiol..

[56] Pitt B., Zannad F., Remme W.J., Cody R., Castaigne A., Perez A., Palensky J., Wittes J. (1999). The effect of spironolactone on morbidity and mortality in patients with severe heart failure. Randomized Aldactone Evaluation Study Investigators. N. Engl. J. Med..

[57] Zannad F., McMurray J.J.V., Krum H., van Veldhuisen D.J., Swedberg K., Shi H., Vincent J., Pocock S.J., Pitt B., EMPHASIS-HF Study Group (2011). Eplerenone in patients with systolic heart failure and mild symptoms. N. Engl. J. Med..

[58] Alexandrou M.-E., Papagianni A., Tsapas A., Loutradis C., Boutou A., Piperidou A., Papadopoulou D., Ruilope L., Bakris G., Sarafidis P. (2019). Effects of mineralocorticoid receptor antagonists in proteinuric kidney disease: A systematic review and meta-analysis of randomized controlled trials. J. Hypertens..

[59] Navaneethan S.D., Nigwekar S.U., Sehgal A.R., Strippoli G.F.M. (2009). Aldosterone antagonists for preventing the progression of chronic kidney disease: A systematic review and meta-analysis. Clin. J. Am. Soc. Nephrol..

[60] McMurray J.J.V., O’Meara E. (2004). Treatment of heart failure with spironolactone—Trial and tribulations. N. Engl. J. Med..

[61] Kolkhof P., Nowack C., Eitner F. (2015). Nonsteroidal antagonists of the mineralocorticoid receptor. Curr. Opin. Nephrol. Hypertens.

[62] Pitt B., Kober L., Ponikowski P., Gheorghiade M., Filippatos G., Krum H., Nowack C., Kolkhof P., Kim S.-Y., Zannad F. (2013). Safety and tolerability of the novel non-steroidal mineralocorticoid receptor antagonist BAY 94-8862 in patients with chronic heart failure and mild or moderate chronic kidney disease: A randomized, double-blind trial. Eur. Heart J..

[63] Bakris G.L., Agarwal R., Chan J.C., Cooper M.E., Gansevoort R.T., Haller H., Remuzzi G., Rossing P., Schmieder R.E., Nowack C. (2015). Effect of Finerenone on Albuminuria in Patients With Diabetic Nephropathy: A Randomized Clinical Trial. JAMA.

[64] Bakris G.L., Agarwal R., Anker S.D., Pitt B., Ruilope L.M., Rossing P., Kolkhof P., Nowack C., Schloemer P., Joseph A. (2020). Effect of Finerenone on Chronic Kidney Disease Outcomes in Type 2 Diabetes. N. Engl. J. Med..

[65] Pitt B., Filippatos G., Agarwal R., Anker S.D., Bakris G.L., Rossing P., Joseph A., Kolkhof P., Nowack C., Schloemer P. (2021). Cardiovascular Events with Finerenone in Kidney Disease and Type 2 Diabetes. N. Engl. J. Med..

[66] Davenport A.P., Hyndman K.A., Dhaun N., Southan C., Kohan D.E., Pollock J.S., Pollock D.M., Webb D.J., Maguire J.J. (2016). Endothelin. Pharm. Rev..

[67] Benigni A. (1995). Defining the role of endothelins in renal pathophysiology on the basis of selective and unselective endothelin receptor antagonist studies. Curr. Opin. Nephrol. Hypertens.

[68] Neuhofer W., Pittrow D. (2009). Endothelin receptor selectivity in chronic kidney disease: Rationale and review of recent evidence. Eur. J. Clin. Investig..

[69] Fenhammar J., Andersson A., Frithiof R., Forestier J., Weitzberg E., Sollevi A., Hjelmqvist H. (2008). The endothelin receptor antagonist tezosentan improves renal microcirculation in a porcine model of endotoxemic shock. Acta Anaesthesiol. Scand..

[70] de Zeeuw D., Coll B., Andress D., Brennan J.J., Tang H., Houser M., Correa-Rotter R., Kohan D., Lambers Heerspink H.J., Makino H. (2014). The endothelin antagonist atrasentan lowers residual albuminuria in patients with type 2 diabetic nephropathy. J. Am. Soc. Nephrol..

[71] Mann J.F.E., Green D., Jamerson K., Ruilope L.M., Kuranoff S.J., Littke T., Viberti G., ASCEND Study Group (2010). Avosentan for overt diabetic nephropathy. J. Am. Soc. Nephrol..

[72] Heerspink H.J.L., Parving H.-H., Andress D.L., Bakris G., Correa-Rotter R., Hou F.-F., Kitzman D.W., Kohan D., Makino H., McMurray J.J.V. (2019). Atrasentan and renal events in patients with type 2 diabetes and chronic kidney disease (SONAR): A double-blind, randomised, placebo-controlled trial. Lancet.

[73] Scirica B.M., Bhatt D.L., Braunwald E., Steg P.G., Davidson J., Hirshberg B., Ohman P., Frederich R., Wiviott S.D., Hoffman E.B. (2013). Saxagliptin and Cardiovascular Outcomes in Patients with Type 2 Diabetes Mellitus. N. Engl. J. Med..

[74] Rosenstock J., Kahn S.E., Johansen O.E., Zinman B., Espeland M.A., Woerle H.J., Pfarr E., Keller A., Mattheus M., Baanstra D. (2019). Effect of Linagliptin vs Glimepiride on Major Adverse Cardiovascular Outcomes in Patients With Type 2 Diabetes: The CAROLINA Randomized Clinical Trial. JAMA.

[75] Tuttle K.R., Brosius F.C., Adler S.G., Kretzler M., Mehta R.L., Tumlin J.A., Tanaka Y., Haneda M., Liu J., Silk M.E. (2018). JAK1/JAK2 inhibition by baricitinib in diabetic kidney disease: Results from a Phase 2 randomized controlled clinical trial. Nephrol. Dial. Transpl..

[76] Chen Y., Lee K., Ni Z., He J.C. (2020). Diabetic Kidney Disease: Challenges, Advances, and Opportunities. Kidney Dis..

[77] Gorin Y., Cavaglieri R.C., Khazim K., Lee D.-Y., Bruno F., Thakur S., Fanti P., Szyndralewiez C., Barnes J.L., Block K. (2015). Targeting NADPH oxidase with a novel dual Nox1/Nox4 inhibitor attenuates renal pathology in type 1 diabetes. Am. J. Physiol. Ren. Physiol..

[78] Cha J.J., Min H.S., Kim K.T., Kim J.E., Ghee J.Y., Kim H.W., Lee J.E., Han J.Y., Lee G., Ha H.J. (2017). APX-115, a first-in-class pan-NADPH oxidase (Nox) inhibitor, protects db/db mice from renal injury. Lab. Investig..

[79] Boels M.G.S., Koudijs A., Avramut M.C., Sol W.M.P.J., Wang G., van Oeveren-Rietdijk A.M., van Zonneveld A.J., de Boer H.C., van der Vlag J., van Kooten C. (2017). Systemic Monocyte Chemotactic Protein-1 Inhibition Modifies Renal Macrophages and Restores Glomerular Endothelial Glycocalyx and Barrier Function in Diabetic Nephropathy. Am. J. Pathol..

[80] Menne J., Eulberg D., Beyer D., Baumann M., Saudek F., Valkusz Z., Więcek A., Haller H., Emapticap Study Group (2017). C-C motif-ligand 2 inhibition with emapticap pegol (NOX-E36) in type 2 diabetic patients with albuminuria. Nephrol. Dial. Transpl..

[81] Tye S.C., Denig P., Heerspink H.J.L. (2021). Precision medicine approaches for diabetic kidney disease: Opportunities and challenges. Nephrol. Dial. Transpl..

